# Novel Antitumor Agents Based on Fluorescent Benzofurazan Derivatives and Mesoporous Silica

**DOI:** 10.3390/ijms232415663

**Published:** 2022-12-10

**Authors:** Madalina Tudose, Daniela C. Culita, Rodica D. Baratoiu-Carpen, Raul-Augustin Mitran, Andrei Kuncser, Cosmin Romanitan, Roxana Cristina Popescu, Diana Iulia Savu

**Affiliations:** 1 Ilie Murgulescu Institute of Physical Chemistry, 202 Splaiul Independentei, 060021 Bucharest, Romania; 2 National Institute of Materials Physics, Atomistilor Street No. 405 A, 077125 Magurele, Romania; 3 National Institute for Research and Development in Microtechnologies, IMT-Bucharest, 126A Erou Iancu Nicolae Street, 077190 Bucharest, Romania; 4 Department of Life and Environmental Physics, Horia Hulubei National Institute for Research and Development in Physics and Nuclear Engineering, Reactorului 30, 077125 Magurele, Romania; 5 Department of Science and Oxide Materials and Nanomaterials, University Politehnica of Bucharest, 1–7 Gheorghe Polizu Street, 011061 Bucharest, Romania; 6 Department of Bioengineering and Biotechnology, Faculty of Medical Engineering, University Politehnica of Bucharest, 1–7 Gheorghe Polizu Street, 011061 Bucharest, Romania

**Keywords:** mesoporous silica, benzofurazan derivatives, fluorescence, antitumor activity

## Abstract

Two novel fluorescent mesoporous silica-based hybrid materials were obtained through the covalent grafting of [4-hydrazinyl-7-nitrobenz-[2,1,3-*d*]-oxadiazole (NBDH) and N^1^-(7-nitrobenzo[*c*][1,2,5]-oxadiazol-4-yl) benzene-1,2-diamine (NBD-PD), respectively, inside the channels of mesoporous silica SBA-15. The presence of fluorescent organic compounds (nitrobenzofurazan derivatives) was confirmed by infrared spectroscopy (IR), X-ray photoelectron spectroscopy (XPS), thermal analysis (TG), and fluorescence spectroscopy. The nitrogen physisorption analysis showed that the nitrobenzofurazan derivatives were distributed uniformly on the internal surface of SBA-15, the immobilization process having a negligible effect on the structure of the support. Their antioxidant activity was studied by measuring the ability to reduce free radicals DPPH (free radical scavenging activity), in order to formulate potential applications of the materials obtained. Cytotoxicity of the newly synthesized materials, SBA-NBDH and SBA-NBD-PD, was evaluated on human B16 melanoma cells. The morphology of these cells, internalization and localization of the investigated materials in melanoma and fibroblast cells were examined through fluorescence imaging. The viability of B16 (3D) spheroids after treatment with SBA-NBDH and SBA-NBD-PD was evaluated using MTS assay. The results showed that both materials induced a selective antiproliferative effect, reducing to various degrees the viability of melanoma cells. The observed effect was enhanced with increasing concentration. SBA-NBD-PD exhibited a higher antitumor effect compared to SBA-NBDH starting with a concentration of 125 µg/mL. In both cases, a significantly more pronounced antiproliferative effect on tumor cells compared to normal cells was observed. The viability of B16 spheroids dropped by 40% after treatment with SBA-NBDH and SBA-NBD-PD at 500 µg/mL concentration, indicating a clear cytotoxic effect of the tested compounds. These results suggest that both newly synthesized biomaterials could be promising antitumor agents for applications in cancer therapy.

## 1. Introduction

Over the past decades, the progress made in developing new classes of nanotechnology-based drugs for the treatment of cancer (of any kind) has led to an increase in the life expectations of millions of cancer patients due to their improved efficacy, safety, and reasonable cost over the existing therapeutic products available in the market [1]. According to the scientific literature, this nanotechnological progress continues to grow at an astonishing rate leading to complex materials and systems with smart proprieties such as biological, disease diagnosis, medical imaging, tissue regeneration, and drug delivery [2,3,4].

From the multitude of functionalized nanomaterials reported in the literature, polymeric liposomes, dendrimers, and self-assembly nanoparticles, or gold inorganic nanoparticles, ferric oxide nanoparticles, carbon nanotubes, mesoporous silica nanoparticles (MSNs), are ideal examples of biocompatible materials whose biochemical applications have been intensively studied [5,6]. Among them, mesoporous silica particles have attracted much attention because of their thermal stabilities, high surface area (ca. 1000 m^2^/g), high pore volumes (ca. 1 cm^3^/g), uniform and tunable pore sizes [7,8,9]. In addition to these properties, high physical stability is one of the important features [7]. An important representative of this type of mesoporous silica nanoparticles (MSNs) is SBA-15 (Santa Barbara Amorphous), whose importance is due to its unique properties, including adjustable pores, mechanical stability, large surface area, and controllable thickness [7]. Given the known properties, the surface engineering of mesoporous silica nanoparticles is one of the most widely used strategies to diversify their functionality and improve their biological behaviors, such as cellular uptake, biodistribution, and immunostimulatory activities [10,11]. In this regard, one of the most important applications of SBA-15 is its use for covalent or non-covalent immobilization of compounds of interest in order to adapt properties such as hydrophilicity or hydrophobicity and reactivity [7].

Although several publications have reported the non-covalent functionalization of mesoporous silica (SBA-15) with different compounds (drugs) [12,13,14], benzofurazans linked to mesoporous silica have not been reported yet. The importance of benzofurazans has been reported in the literature since 1968, being known for their bactericidal, fungicidal, insecticidal activity and, also, inhibitory effects on the biosynthesis of nucleic acids and proteins in various mammals [15]. Their fluorescent properties have also been investigated by many researchers since 1991 [16,17,18]. All these properties, and the demonstrated antiproliferative activity (in vitro and in vivo) of the benzofurazan compounds against several neoplastic cell lines [15], determined us to develop fluorescent mesoporous hybrid biomaterials through the immobilization of some benzofurazan derivatives on the surface of SBA-15 mesoporous silica and to investigate their cytotoxic activity. The unique features of SBA-15 such as highly ordered pore structure, large specific surface area, and high pore volume are expected to allow high loading of poorly water-soluble benzofurazan derivatives, to increase their solubility and bioavailability, and also to limit their unwanted side effects on healthy cells. Moreover, the SBA-15 mesoporous silica that functions as a drug carrier in the hybrid materials can prevent the drug’s metabolism and clearance, providing it with better pharmacokinetics than the free drug. In many of the hybrid systems reported so far, it was revealed that the carrier itself had no therapeutic effect, but provided enhanced efficacy and/or reduced drug toxicity [19,20,21]. All these aspects made us believe that the hybrid materials that we propose to obtain can be superior in terms of their biological activity compared to the individual components.

Therefore, in this study, we present the synthesis, physicochemical characterization, and biological evaluation (antioxidant and cytotoxic activity in melanoma cells) of two novel composites based on [4-hydrazinyl-7-nitrobenz-[2,1,3-d]-oxadiazole] (NBDH) and N^1^-(7-nitrobenzo[c][1,2,5] oxadiazol-4-yl)-benzene-1,2-diamine (NBD-PD) grafted on SBA-15 mesoporous silica. As far as we know, this is the first study in the literature regarding the development and bioevaluation of these types of hybrid materials.

## 2. Results and Discussion

### 2.1. Synthesis of Compounds

**Scheme 1:**
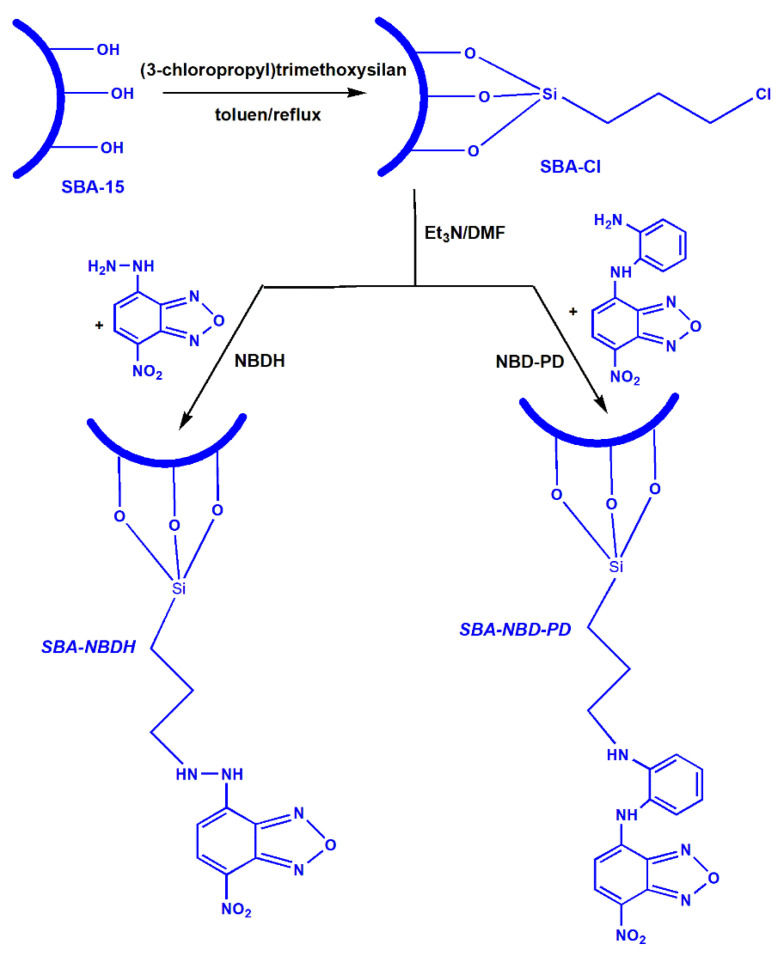
Schematic protocol for functionalization of SBA-15 with NBD derivatives.

In this manuscript, we present an easy method to obtain two new materials starting from mesoporous silica SBA-15 and two nitrobenzofurazan derivatives, which are known in the literature as having antimicrobial and anticancer effects [15]. The process of synthesis of SBA-NBD derivatives takes place through the reaction between NBDH/NBD-PD and SBA-Cl, according to Scheme 1. This process contains two steps: the first one consists of the reaction of SBA-15 with (3-chloropropyl)trimethoxysilane (CPTES), and the second one, the reaction of nitrobenzofurazan derivatives with SBA-Cl [7] leading to the new materials presented and further characterized.

### 2.2. Characterization of Materials

The synthesized materials were characterized by FT/IR spectroscopy, N_2_ adsorption-desorption analysis, electron microscopy (SEM/TEM), fluorescence spectroscopy, X-ray photoelectron spectroscopy (XPS), small-angle X-ray scattering (SAXS) and, thermal analysis. All the characterization methods highlighted the functionalization of SBA-15 silica with NBD derivatives.

**Figure 1:**
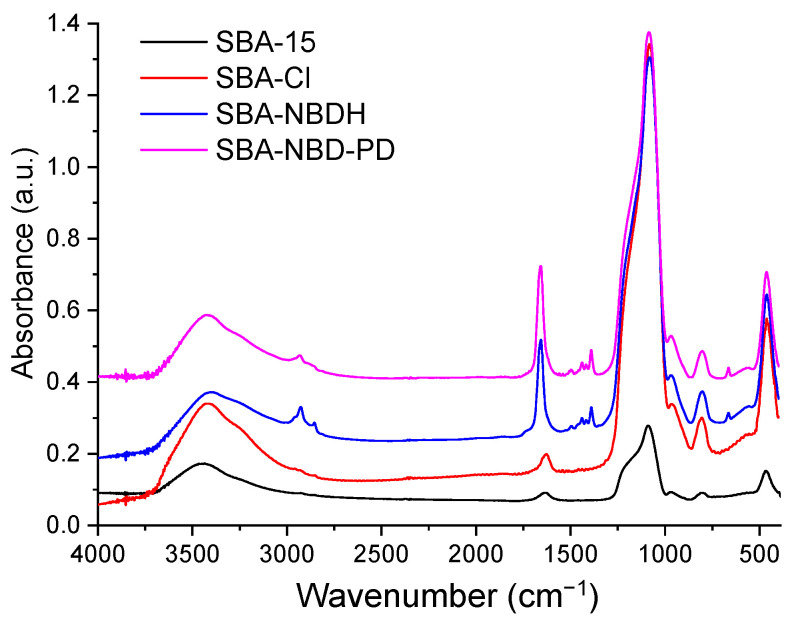
FTIR spectra of pure SBA-15, SBA-Cl, SBA-NBDH, and SBA-NBD-PD.

Figure 1 presents the FTIR spectra of the pure SBA-15, SBA-Cl, and NBD-functionalized SBA-15 samples. All spectra show bands at 1082 and 805 cm^−1^ which are attributed to asymmetric and symmetric Si-O-Si stretching vibrations of the mesoporous silica framework, 970 cm^−1^ corresponding to Si–OH stretching vibrations, and 460 cm^−1^ corresponding to symmetric Si–O bending vibration [22,23]. The broad band centered at ~3400 cm^−1^ and that at 1630 cm^−1^ are characteristic of the stretching vibrations of hydroxyl groups of absorbed water molecules and bending vibrations of Si–OH, respectively [24]. The peaks at 2927 and 2855 cm^−1^ that appear in the spectra of chloropropyl-SBA, SBA-NBDH and SBA-NBDPD are due to CH aliphatic vibrations groups. New bands can be observed in the spectra of SBA-NBDH and SBA-NBD-PD, in the region 1660–1390 cm^−1^, associated with the functional groups of the nitrobenzofurazan derivatives. Excepting the band centered at ~1656 cm^−1^ of medium intensity, the other bands have low intensities, being hampered by the very intense bands of silica. The presence of these new bands evidences the successful grafting of the nitrobenzofurazan derivatives on the silica surface.

**Figure 2:**
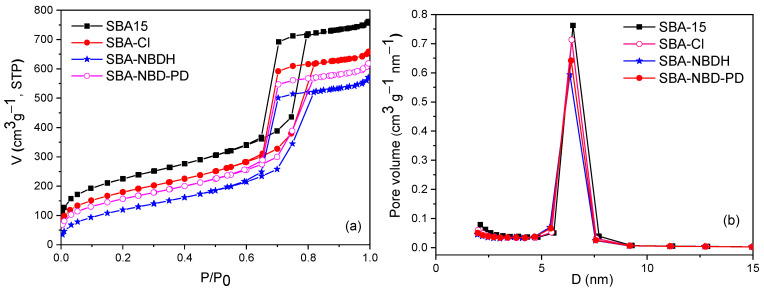
N_2_ adsorption–desorption isotherms (**a**) and pore size distribution graphs (**b**) of the samples.

N_2_ adsorption–desorption isotherms (Figure 2a) were recorded on pristine SBA-15, SBA-Cl, and the NBD-functionalized SBA-15. All four isotherms are of type IV with an H1 hysteresis loop, according to IUPAC classification [25], typical for mesoporous materials with regular pore structure. The textural parameters (BET surface area, total pore volume, and average pore size) are listed in Table 1. The decrease in pore volumes and BET surface areas of SBA-Cl and functionalized SBA-15 samples in comparison with those of the pure SBA-15 can be attributed to the presence of functional groups on the internal walls of pore channels that partially obstruct the adsorption of nitrogen. The physisorption data indicate a uniformity of the functional groups’ distribution and a preservation of the regularity of the mesoporous structures after functionalization. Figure 2b indicates that the pore size distribution for the NBD-functionalized samples is very close to that of the SBA-15 sample, the peak maxima being slightly shifted to lower values. However, the average pore size values are similar, as can be seen in Table 1.

**Figure 3:**
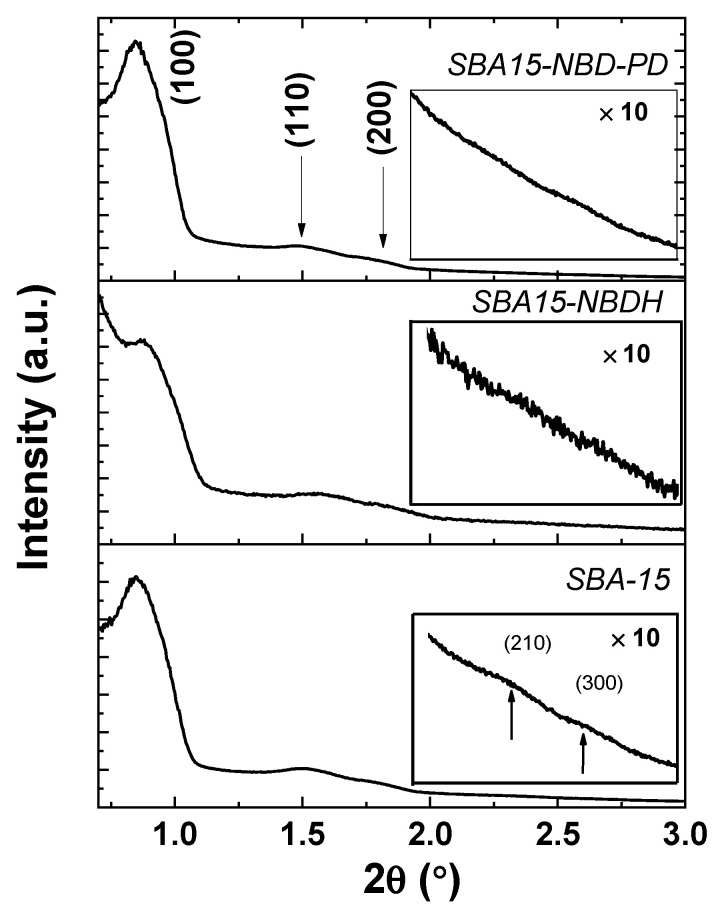
Small angle XRD patterns of SBA-15, SBA-NBDH, and SBA-NBD-PD.

Small angle X-ray diffraction (XRD) patterns were recorded in θ/2θ mode, from 0.7 to 3° with a step of 0.0004° and speed of 0.48°, respectively (Figure 3). Each XRD pattern exhibits three diffraction features located at 0.89, 1.49, and 1.79°, which correspond to the (1 0 0), (1 1 0), and (2 0 0) scattering reflections of two-dimensional hexagonal p6mm pore structure, which are akin to those of conventional SBA-15 [26]. In addition, at larger magnification (e.g., ×10), (201), and (300) scattering reflections become visible for the SBA-15 sample, indicating that the as-synthesized pure SBA-15 has a high degree of hexagonal mesoscopic organization than the other samples [27]. Further, the interplanar distance was calculated using Bragg’s law which relates its value to the angular position [28]. The values of the interplanar distances are listed in Table 2.

Similar interplanar distances for mesoporous silica were also reported by Zhao et al. [27]. Usually, the interplanar distance on the (100) atomic plane in these structures is ascribed to the pore-to-pore center correlation distance [29]. Regarding the crystal quality, the size of the crystalline domains (calculated by the Debye–Scherrer equation) [30] was not significantly altered after functionalization with nitrobenzofurazan derivatives (~30 nm in each case).

**Figure 4:**
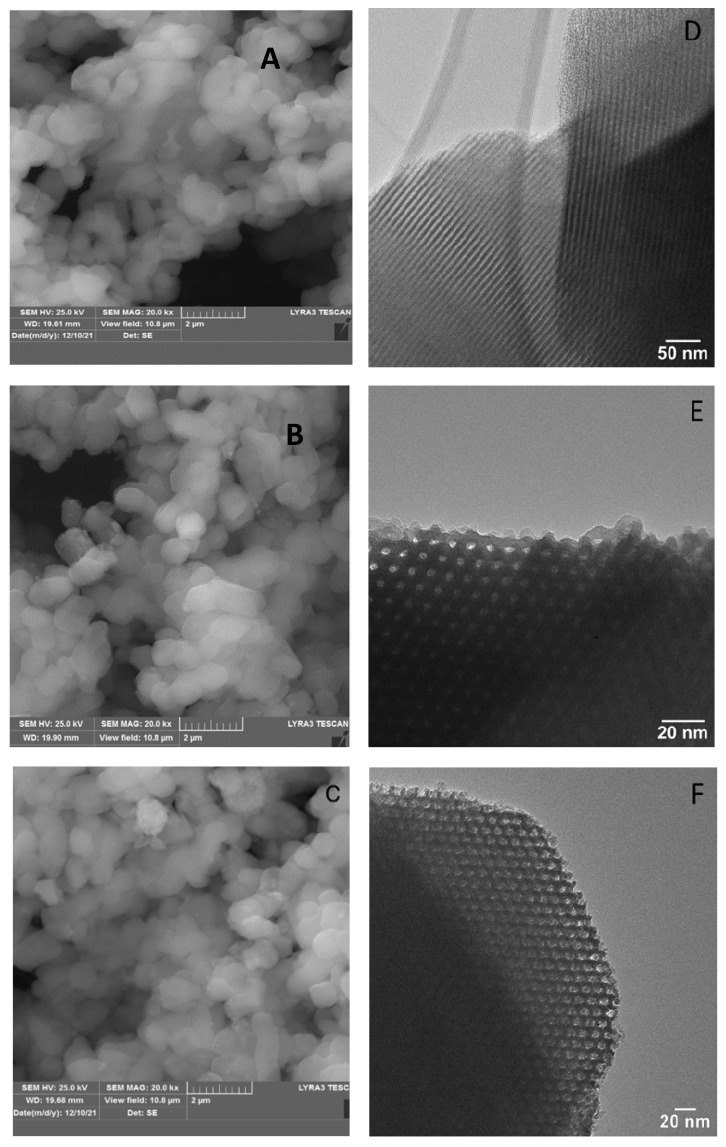
SEM images of SBA-15 (**A**), SBA-NBDH (**B**), and SBA-NBD-PD (**C**) and TEM images of SBA-15 (**D**), SBA-NBDH (**E**), and SBA-NBD-PD (**F**).

SEM images illustrate the SBA-15 and functionalized SBA-15 samples show uniform particles of ~1 μm with the same morphology (Figure 4A–C). TEM micrographs (Figure 4D–F) reveal in all cases the ordered hexagonal channels specific to mesoporous SBA-15.

**Figure 5:**
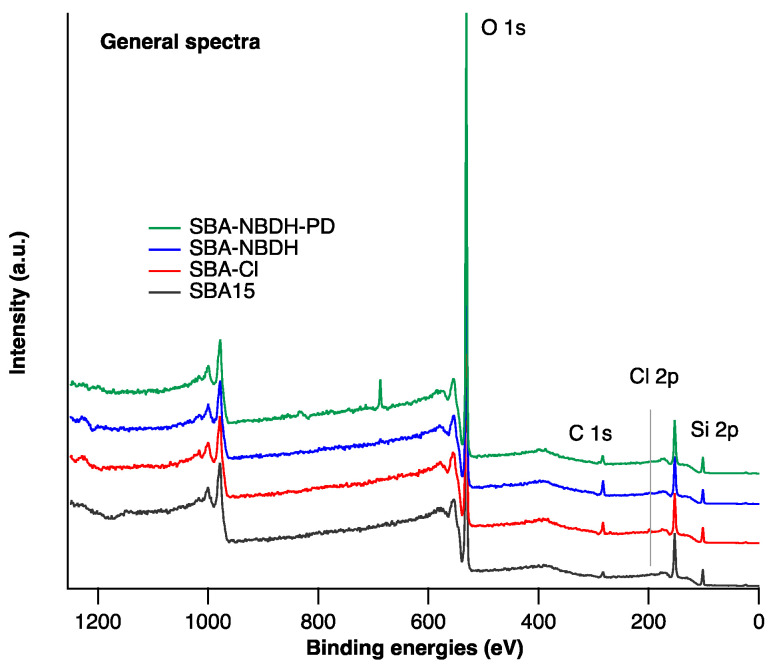
XPS general spectra of studied samples.

Figure 5 gathers the general XPS spectra of SBA-Cl, SBA-NBDH, and SBA-NBD-PD samples. By analyzing these spectra, it can be seen that the SBA15 matrix has been functionalized. First, the chlorine-specific signal for the SBA-Cl sample is observed at ~200 eV, which means that the chlorine atoms have been attached to the SBA15 matrix. For SBA-NBDH and SBA-NBD-PD samples, the appearance of the N1s signal at ~400 eV is observed after the reaction of nitrobenzofurazan with Cl atoms, which are no longer observed on the surface.

The compositional analysis of the surface of all samples shows an increase in the percentage of C after SBA-Cl surface modification, which confirms once again the functionalization of these materials (Table 3) [31].

**Figure 6:**
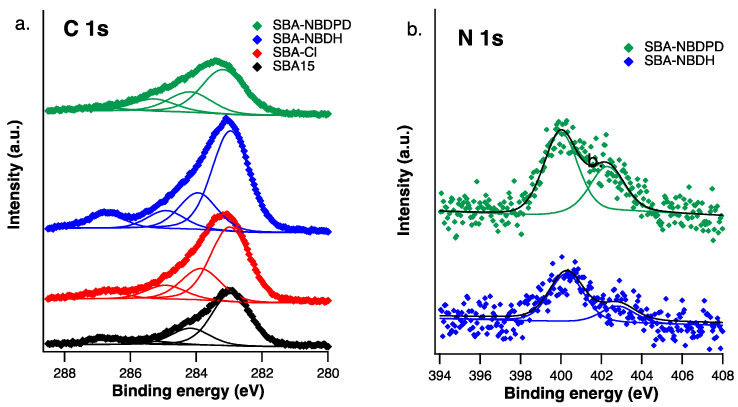

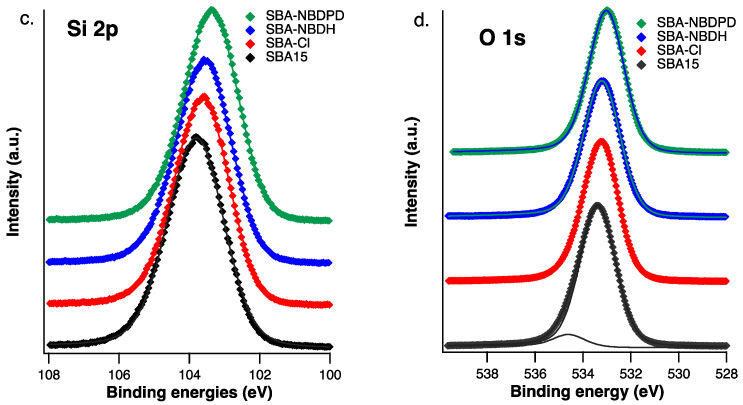
High-resolution spectra for C1s (**a**), N1s (**b**), O1s (**c**), and Si 2p (**d**) core levels.

Analysis of high-resolution C1s spectra (Figure 6a), shows for C1s the composition of the contaminating carbon for SBA-15, with specific *sp^3^*C (~284.6 eV), *sp^2^*C (~285.5 eV), C-O (~286.6 eV) and C=O signals at (~288.3 eV). Upon functionalization, a slight increase in the *sp^2^*C component and of the component at ~286.6 eV (associated with the C-N bond) is observed, due to nitrobenzenfurazan derivatives presence. Deconvolution analysis of N1s spectra (Figure 6b) shows two components, one at ~400.1 eV associated with *sp^2^* C-N bondings and a second component at 402.5 eV corresponding to N-O bondings [32]. The ratio between the two components is ~3:1 (C-N/N-O).

Si 2p signal found at ~103.3 eV is specific for the Si-O bond in SBA-15. Upon functionalization the binding energy shifts to lower values most probably due to the formation of the Si-C bond. O1s found at ~533.3 eV corresponds to O^2-^ ions from the matrix, while a small component associated with the hydroxyl group can be always observed at ~534.6 eV for the SBA-15 sample (Figure 6c,d). After the functionalization procedure, the OH^−^ groups disappear and the O^2−^ ions slightly shift to lower binding energies due to the formation of new O-Si-C bonds.

**Figure 7:**
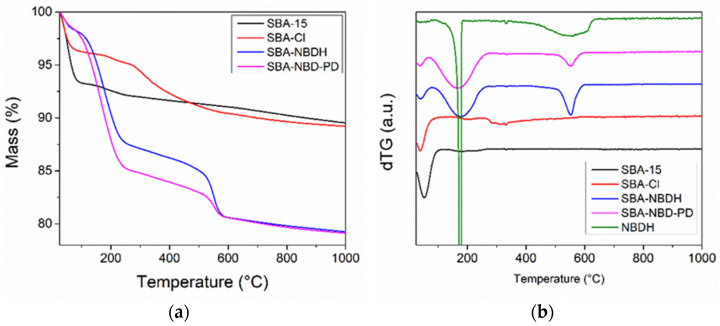
Thermogravimetric analyses (**a**) and differential thermogravimetric analyses (dTG) (**b**) of the pristine and functionalized SBA-15 matrices.

The efficiency of the functionalization of SBA-15 has been evaluated by using the results obtained from the thermogravimetric analysis. Thermogravimetric analyses (TGA) were carried out in order to quantitatively assess the number of organic groups present in the functionalized SBA-15 samples (Figure 7), [33]. All samples show a 2–7% wt. mass loss between 25 and 100 °C, which is ascribed to the desorption of water and residual solvents (Figure 7a). The pristine SBA-15 matrix shows a gradual 3.9% wt. mass loss with respect to dry samples between 110 and 1000 °C, which could be explained by the dehydration of surface silanol groups and residual carbon. An additional mass loss event can be noticed between 240–600 °C for SBA-Cl, indicating the successful functionalization with chloropropyl groups. This additional mass loss corresponds to a 3.6% wt. chloropropyl content, or a molar ratio SiO_2_:-C_3_H_6_Cl = 1:0.029. Both SBA-NBDH and SBA-NBD-PD exhibit two mass loss events, centered around 175 °C and 550 °C, respectively. The two-step combustion of the nitrogen-containing groups is similar to that of pure NBDH (Figure 7b). Molar ratio SiO_2_: NDBH/NBD-PD = 1:0.051 and 1:0.040 were obtained, respectively, indicating the complete substitution of the chloropropyl group. The slight molar NBDH excess versus chloropropyl in the case of SBA-NBDH could be explained by additional NDBH adsorption onto the silica surface.

**Figure 8:**
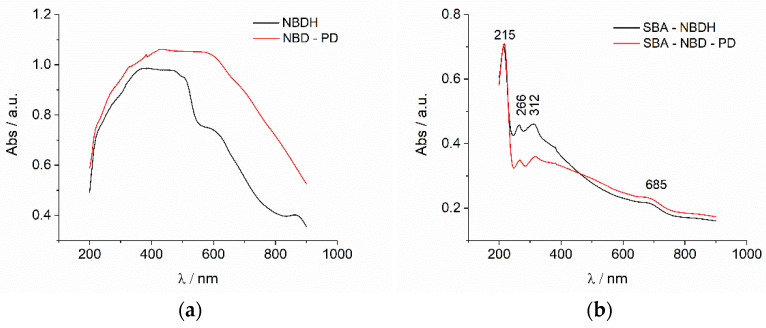
Absorption spectra of NBDH/NBD-PD (**a**) and SBA-NBDH/SBA-NBD-PD (**b**).

Fluorescence study represents another method used for evidencing the forming of new materials. To characterize the grafting process of NBDH /NBD-PD by chemically functionalized SBA-15, Figure 8 shows by comparison the absorption spectra of SBA-NBDH/SBA-NBD-PD and NBDH/NBD-PD. A broad absorption band with a maximum at ~420 nm for NBDH is observed, whereas, for NBD-PD, the absorption maximum is slightly bathochromic shifted to ~500 nm, both of them corresponding to the absorption of the NBD group (Figure 8a). After being grafted into the channel of SBA-15, well-structured absorption peaks, at 215, 266, and 312 nm corresponding to typical electronic transitions of the aromatic ring are attributed to SBA-15 absorption. The broad but less intense band between 400 and 600 nm that can be observed in the absorption spectra of SBA-NBDH/SBA-NBD-PD may sustain the grafting of NBD derivatives on the SBA-15 surface (Figure 8b). The band at 685 nm is due to impurities in the SBA-15 structure.

**Figure 9:**
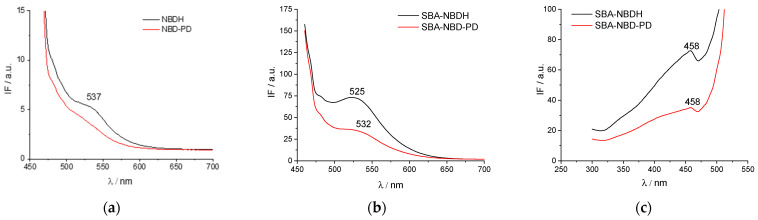
Fluorescence emission spectra of NBDH and NBD-PD (**a**), of SBA-NBDH and SBA-NBD-PD (**b**), λ_ex_ = 450 nm, and fluorescence excitation spectra of SBA-NBDH and SBA-NBD-PD (**c**), λ_em_ = 530 nm.

Fluorescence emission spectra of NBDH and NBD-PD compared to those of SBA-NBDH and SBA-NBD-PD, at λ_ex_ = 450 nm, are shown in Figure 9a,b. The fluorescence emission of NBDH appears at λ_em_ = 537 nm and corresponds to the NBD group, this being quite small due to –NH–NH_2_ substitution from the 8 position of the NBD group, and totally quenched for NBD-PD, due to 1,2-diamine (Figure 9a) [15]. After being grafted into the channels of SBA-15, the fluorescence emission of both compounds increases and records a significant blue-shift of 12 nm for SBA-NBDH, at λ_em_ = 525, and of 5 nm for SBA-NBD-PD, at λ_em_ = 532 nm (Figure 9b). According to Zhang et al. [34], this feature may be corroborated with the molecular orbital confinement theory that all energy levels of guest molecules increase in the channel of the host as a result of the confinement [34]. Also, it can be seen that the fluorescence emission of SBA-NBDH is two orders of magnitude higher than that of SBA-NBD-PD and is about 15 times more intense than that of NBDH, before being grafted into the channels of SBA-15. This enhancement of the fluorescence emission is due to open pores on the SBA-15 surface, as well as to the uniformity of the SBA-15 surface containing these pores, therefore to the SBA-15 surface functionalization. Fluorescence excitation spectra of SBA-NBDH and SBA-NBD-PD showed one major peak at ~458 nm, which corresponds to NBD absorption when grafted on the SBA-15 surface, so that on the excited state of porous NBDH and NBD-PD grafted SBA-15, no significant damage for the NBD group, occurs (Figure 9c).

Overall, for NBDH/NBD-PD chemically functionalized SBA-15 silica, an enhancement of the fluorescence emission of SBA-NBDH with λ_em_ = 525 nm as well as of SBA-NBD-PD, with λ_em_ = 532, is noticed. Results are compared with data obtained for NBDH/NBD-PD chemically functionalized graphene oxide, where the fluorescence quenching was observed [15] and is relevant to better understand the biological properties of various NBD-containing systems.

### 2.3. Antioxidant Activity of the SBA-15-NBD Derivatives

**Figure 10:**
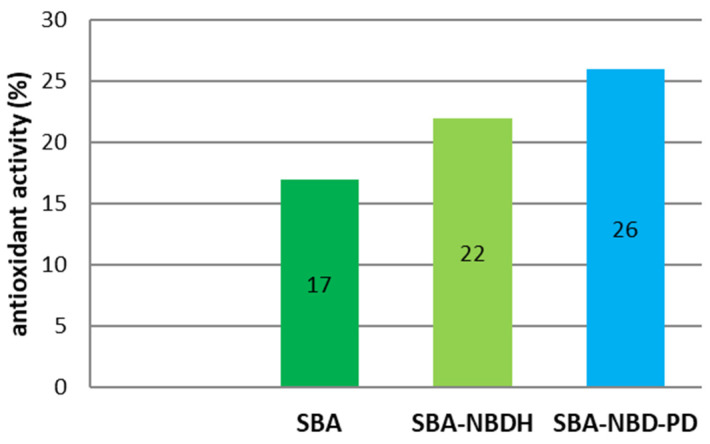
Antioxidant activity by DPPH assay of the SBA-15 with NBD derivatives.

The easiest method known in the literature for determining the antioxidant activity of some molecules of interest is based on measuring the ability to reduce free radicals DPPH (1,1-diphenyl-2-picrylhydrazil) [35]. The experiment consists of the reaction of the antioxidant with the free radical DPPH (purple solution) and which by hydrogen donation turns into a clear DPPH-H, in which the degree of discoloration indicates the antioxidant potential [35]. In our case, the SBA-15 exhibited a lower free radical scavenging effect (17%), as compared to those of SBA-NBDH (22%) and SBA-NBD-PD (26%) (Figure 10). Increased antioxidant activity was observed in the case of SBA-NBD-PD (Figure 10) compared with SBA-15 and SBA-NBDH. Therefore, the functionalization of SBA-15 with NBD derivatives led to an increase in the antioxidant capacity of the novel composites, proving their potential for future applications.

### 2.4. Biological Activity Results

#### 2.4.1. SBA-15-NBD Derivatives Induced a Selective Antiproliferative Effect in Melanoma Cells

**Figure 11:**
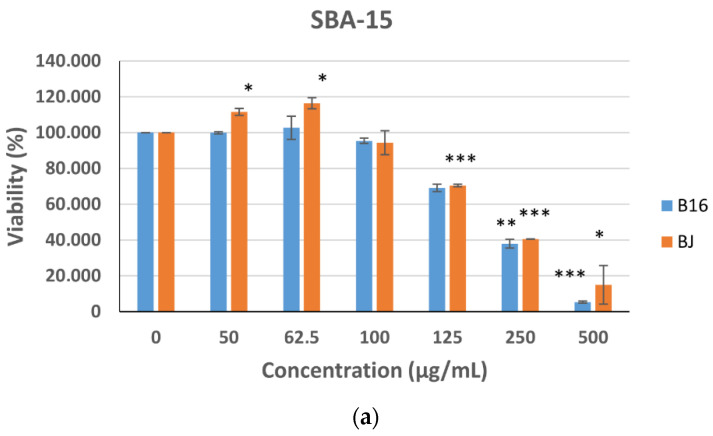

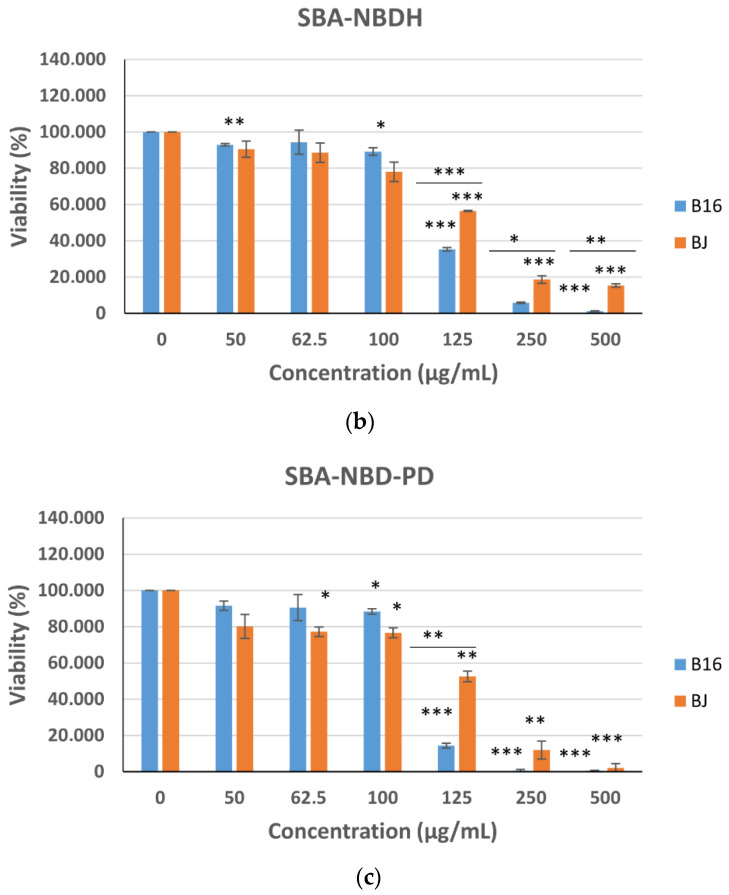
Cytotoxicity of nanoparticles on B16 and BJ cells. Proliferation kinetics of tumor and normal cells at 5 days after exposure to different concentrations of SBA-15 (**a**), SBA-NBDH (**b**), and SBA-NBD-PD (**c**). Data are presented as percentage of untreated control and are shown as mean ± SEM (n = 3). * *p* < 0.05; ** *p* < 0.01; *** *p* < 0.001.

MTT viability results measured at 5 days after treatment showed that both normal and tumor cell viability decreases with the compounds concentration (Figure 11). SBA-15 showed a biocompatible behavior (viability ≥ 70%) for both normal and tumor cells up to the concentration of 125 µg/mL. This compound induced even a stimulating effect on normal BJ cells at 50 µg/mL and 62.5 µg/mL, as their metabolism was highly accentuated after receiving this treatment at all concentrations. The following two concentrations (250 µg/mL and 500 µg/mL) determined cytotoxic effects in both cell types. For SBA-NBDH, the viability of both normal and tumor cells decreases with concentration. In the range of 125–500 µg/mL, a highly cytotoxic effect was observed, with the viability decreasing to under 70%. A significantly more pronounced antiproliferative effect on tumoral cells compared to normal cells was also observed for this range of concentrations (*p* < 0.001 at 125 µg/mL, *p* < 0.05 at 250 µg/mL, *p* < 0.01 at 500 µg/mL). Therefore, SBA-NBDH had a cytotoxic effect on B16 melanoma cells with an IC50 value of 120.12 µg/mL, while for normal cells, the IC50 value was 138.63 µg/mL. A similar behavior was observed in the case of SBA-NBD-PD which induced a higher antitumor effect starting with the concentration of 125 µg/mL. These results are reflected by an IC50 value of 114.11 µg/mL against melanoma cells and an IC50 value of 136.13 µg/mL against fibroblasts. The lower values of IC50 determined in the case of tumor cells indicated that both compounds are more toxic to melanoma cells than to normal skin cells. The obtained selectivity indexes which are higher than 1 (namely, 1.15 in the case of SBA-NBDH and 1.19 in the case of SBA-NBD-PD) reinforced this conclusion.

Analyzing the IC50 values and selectivity indexes obtained (Table 4), it can be inferred that the SBA-NBDH and SBA-NBD-PD compounds are efficient in exhibiting antitumor and antiproliferative effects (SI > 1).

#### 2.4.2. Evaluation of Cell Morphology and Conjugated Nanoparticles Internalization

**Figure 12:**
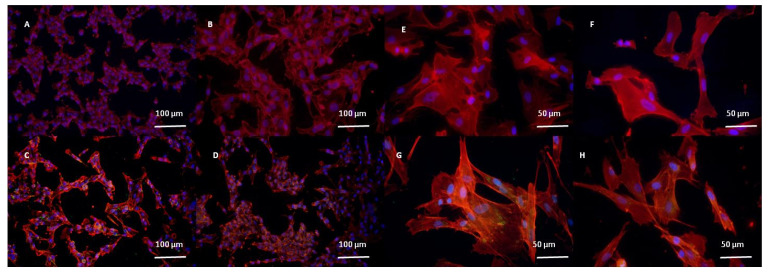
Fluorescence microscopy images of cells at 5 days of culture after the presence/absence of nanoparticles for 16 h: (**A**,**E**) control B16 cells and respectively BJ cells (0 nanoparticles), (**B**,**F**) B16 cells and respectively BJ cells exposed to 62.5 µg/mL SBA-15, (**C**,**G**) B16 cells and, respectively, BJ cells exposed to 62.5 µg/mL to SBA-NBDH, (**D**,**H**) B16 cells and, respectively, BJ cells exposed to 62.5 µg/mL SBA-NBD-PD. Red: Texas Red Phalloidin, blue: Hoechst, green: benzofurazan derivatives autofluorescence (NBDH and NBD-PD).

The morphological investigation, internalization, and localization of nanoparticles in melanoma and fibroblast cells (Figure 12) were performed using fluorescence imaging. The images showed no morphological modification or cytoskeleton alteration (actin filaments maintained their fibril structure) of both normal and tumor cells after their exposure to SBA-15 (Figure 12B,F), SBA-NBDH (Figure 12C,G) and SBA-NBD-PD (Figure 12D,H) compounds with a concentration of 62.5 µg/mL. SBA-NBDH and SBA-NBD-PD could be observed localized in the cytoplasm and in the peri-nuclear area of both tumor (Figure 12C,D) and normal cells (Figure 12G,H).

#### 2.4.3. SBA-15-NBD Derivatives Induced a Reduction of Melanoma Clonogenic Survival

**Figure 13:**
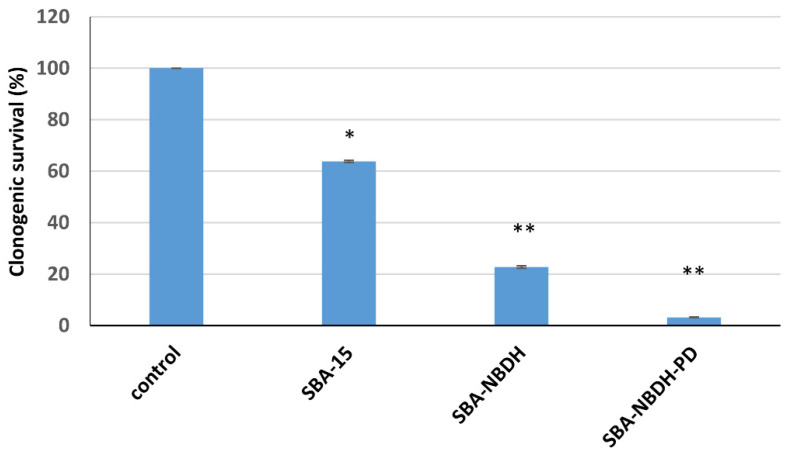
Clonogenic survival of B16 cells seeded in the colony formation assay after exposure to 125 μg/mL SBA-15, SBA-NBDH, and SBA-NBD-PD for 16 h. Data are presented as percentage of untreated control and are shown as mean ± SEM (n = 3). * 0.01 < *p* < 0.05, ** 0.001 < *p* < 0.01.

Long-term survival effects on B16 melanoma cells (Figure 13) were investigated for the equivalent concentration of 125 µg/mL, which was proved to be cytotoxic in the MTT assay. At 10 days after seeding, the cell survival showed a high cytotoxic effect of SBA-NBDH and SBA-NBDH-PD, which showcased a survival lower than 20%, while SBA-15 seemed to be less affected, as the clonogenic survival is around 63%. These outcomes suggest that by binding NBD derivatives on the surface of SBA-15, an enhanced cytotoxic effect is achieved, as demonstrated using the MTT assay.

#### 2.4.4. Tumor Cells Undergo Mitotic Catastrophe after Exposure to Nanoparticles

The use of DNA-damaging agents stimulates different types of cell death including mitotic catastrophe, enhancing the therapeutic effect. Mitotic catastrophe was often described [36,37] as the capacity of cells displaying DNA damage to enter mitosis without attaining DNA repair in G2.

**Figure 14:**
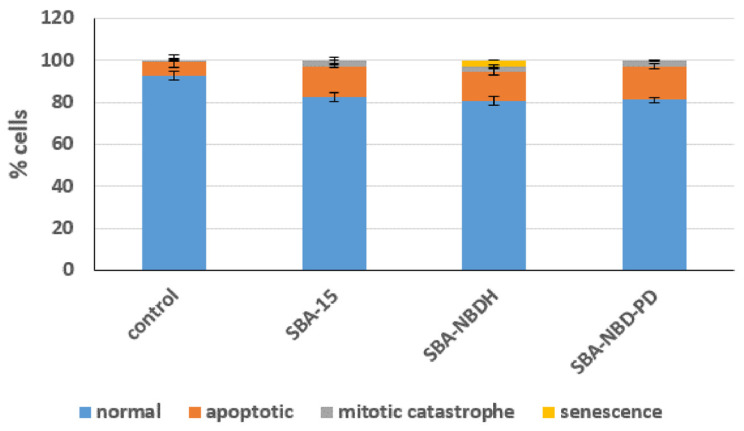
Cells death induced by 125 g/mL nanoparticles, SBA-15, SBA-NBDH, SBA-NBD-PD, in B16 cells. Cells were treated for 16 h and after 5 days subjected to morphological evaluation and classification as apoptotic, multinuclear, or senescent. Data are presented as mean ± SEM.

Morphological analysis of the types of cell death (i.e., apoptosis, mitotic catastrophe, senescence) was investigated at 5 days in B16 tumor cells after the treatment with 125 µg/mL nanoparticles (SBA-15, SBA-NBDH, and SBA-NBD-PD). The results depicted in Figure 14 showed that all the materials induced a statistically significant level of apoptosis (14.5% ± 0.7%, *p* < 0.05 for SBA-15-treated cells, 14% ± 1.4%, *p* < 0.05 for SBA-NBDH-treated cells and 16.1% ± 1.2%, *p* < 0.01 for SBD-NPD-PD-treated group of cells, compared to non-treated control). In addition, a significant increase of 6-fold, 5-fold, and 5.8-fold of multi-nucleated cells indicating mitotic catastrophe type of cell death was observed for the cells treated with SBD-15, SBD-NBDH and respectively SBD-NBD-PD (*p* < 0.5, *p* < 0.01, and respectively *p*< 0.05 compared to non-exposed control). A small proportion (3%) of senescent cells was observed only in the case of melanoma cells exposed to the SBA-NBDH compound.

#### 2.4.5. Benzofurazan Derivates Linked to SBA-15 Induced a Cytotoxic Antitumoral Effects in Melanoma Spheroids

**Figure 15:**
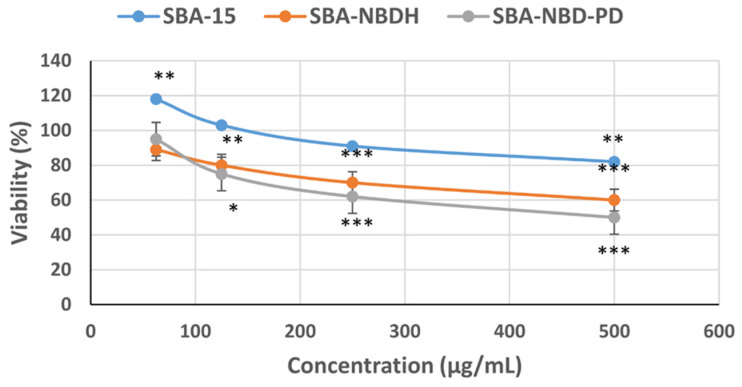
Cytotoxic, antiproliferative effects of SBA-15, SBA-NBDH, and SBA-NBD-PD on B16-spheroids. Spheroids were obtained after seeding 10,000 cells/well and allowed to grow for 3 days and then treated 5 days with different concentrations of silica compounds. Data are presented as mean ± SEM.* 0.01 < *p* < 0.05, ** 0.001 < *p* < 0.01, *** *p* < 0.001.

In addition to a two-dimensional (2D) model for B16 melanoma cells, we employed a three-dimensional (3D) spheroid tumor model, which better mimics in vivo conditions of the intratumoral space [38], in order to test our new materials. Therefore, the viability of B16 spheroids following the treatment with SBA-15, SBA-NBDH, and SBA-NBD-PD was evaluated using MTS assay at 5 days (Figure 15). All the compounds decreased the viability of melanoma spheroids with increasing concentrations. SBA-15 induce a slight reduction of the melanoma spheroids viability (not higher than 20% for the highest concentration), while both benzofurazan derivates had a clear cytotoxic effect induced a drop of the viability of around 40% for the highest concentration (500 µg/mL) used in the experiments.

These outcomes strengthen our previous conclusions that recommend the new SBA-15-NBD derivatives as good potential antitumor biomaterials.

## 3. Materials and Methods

### 3.1. Materials

Tetraethyl orthosilicate ≥ 99.0% (TEOS), (3-chloropropyl)trimethoxysilane 95%, hydrochloric acid (37%), ethyl alcohol, chloroform, and acetonitrile were purchased from Merck Millipore (Darmstadt, Germany). Dry toluene ≥99.5% (≤50 ppm H_2_O) was purchased from Carl Roth (Karlsruhe, Germany). P123 (EO_20_PO_70_EO_20_), 4-chloro-7-nitrobenzofurazan, and 1,2-phenylenediaminewere were supplied by Aldrich (St. Louis, MO, USA).

### 3.2. Characterization Methods

Thermogravimetric analyses (TGA) were performed using a Mettler Toledo TGA/SDTA851e thermogravimeter, at a heating rate of 10 °C min^−1^, in open alumina crucibles and under 20 mL min^−1^ synthetic air flow. N_2_ sorption isotherms were recorded at −196 °C using a Micromeritics ASAP 2020 analyzer. The samples were measured after degassing at 100 °C overnight under vacuum. Specific surface areas (S_BET_) were determined from the linear part of the Brunauer–Emmett–Teller (BET) plots, using adsorption data in the relative pressure range between 0.05 and 0.30. The pore size distribution curves were obtained using the Barrett–Joyner–Halenda (BJH) method from the desorption branch. The total pore volume (V_total_) was determined from the amount adsorbed at the relative pressure of 0.99. Infrared spectra were recorded on a Jasco FT/IR-4700 spectrophotometer. Powder samples were prepared in KBr pellets for IR analysis. In order to assess the structural features (i.e., crystallinity, interplanar distances, pore structure) a 9 kW rotating anode X-ray diffraction system from Rigaku Smart Lab, Japan, was used. During the measurements, Cu Kα1 monochromatic radiation (λ = 1.5406 Å) was used at the nominal power of 40 kV and 75 mA. Microstructural studies were carried out in a TESCAN LYRA 3 XMU SEM-FIB with field emission gun (FEG) and JEOL 2100 with a high-resolution polar piece. UV–vis absorption spectra were performed with a Perkin Elmer Lambda 35 Spectrometer equipped with an integrating sphere, using a spectral one as a certified reflectance standard, at a scanning speed of 60 nm/min and a slit of 4 nm. Fluorescence emission and excitation spectra were registered using the Jasco FP-6500 spectrofluorometer, equipped with EFA-383 epifluorescence attachment, at a scanning speed of 100 nm/min and an excitation wavelength of 450 nm. A Kratos Ultra DLD setup (Kratos Analytical Ltd., Manchester, UK) was used for XPS analysis. It uses a monochromatic Al-Kα source (hν = 1486.74 eV, X-ray source) and a charge neutralizer. All analyses were performed at a pressure of 1 × 10^−7^ Pa and a power of 240 W (20 kV × 12 mA). Casa XPS software was used for spectrum analysis and all samples were calibrated after C1s at 284.6 eV.

### 3.3. Synthesis of the Materials

#### 3.3.1. Synthesis of SBA-15

SBA-15 mesoporous silica was synthesized according to a previously reported method [22], in which 4.0 g of P123 was dissolved under stirring in 124 mL of 2M HCl solution. Afterwards, 8.4 g TEOS was added and the mixture was stirred at 40 °C for 20 h, then aged statically at 100 °C for 48 h. The white solid product was recovered by centrifugation, washed thoroughly with distilled water, dried at 70 °C for 12 h, and calcined at 550 °C for 6 h with a heating rate of 1 °C/min.

#### 3.3.2. Synthesis of SBA-15-Cl

Briefly, 1.26 mL of (3-chloropropyl)trimethoxysilane (CPTES) was added dropwise to a suspension of 0.64 g SBA-15 (previously dried) in 32 mL of anhydrous toluene under stirring, then the mixture was refluxed for 6 h. Afterwards, the solid was separated by centrifugation, washed with toluene and ethyl alcohol, and then dried in an oven at 55 °C [7]. The obtained solid denoted SBA-Cl was certified by various characterization methods and then used in the following syntheses.

#### 3.3.3. Synthesis of [4-hydrazinyl-7-nitrobenz-[2,1,3-d]-oxadiazole (NBDH)

Briefly, 0.2 g of 4-chloro-7-nitrobenzofurazan was dissolved in chloroform, and then the hydrazine solution was added over it and stirred for 1 h at room temperature [15,39]. The reaction product, a yellow-brown solid, was certified by NMR analysis and marked with NBDH [15].

#### 3.3.4. Synthesis of N1-(7-nitrobenzo[c] [1,2,5] oxadiazol-4-yl) benzene- 1,2-diamine (NBD-PD)

Briefly, 0.4 g of 4-chloro-7-nitrobenzofurazandissolved in acetonitrile was poured dropwise into a solution of 1,2-phenylenediamine (0.216 g dissolved in 12 mL acetonitrile). The mixture was stirred for 3 h at room temperature and left to rest until the next day. The processing protocol was the same as reported in the literature [40]. After the reaction mixture was washed with water, filtered, and finally, the solvent evaporated by drying, the solid product was purified on silica gel plates (dichloromethane: methanol). The resulting solid (NBD-PD) was certified by NMR analysis [15].

#### 3.3.5. Synthesis of SBA-15 Functionalized with Nitrobenzofurazan Derivatives

Briefly, 0.2 g of SBA-Cl was suspended in 15 mL of dimethylformamide (DMF), and then 0.08 g of NBD-PD and 100 μL triethylamine (Et_3_N) were added under vigorous stirring. The whole mixture was stirred and refluxed for 24 h. After refluxing, it was left to stand at room temperature for another 24 h, then centrifuged, washed with DMF and ethanol, and dried at 65 °C in air (24 h). The resulting composite was noted as SBA-NBD-PD. The same protocol was adopted for the functionalization of SBA-15 with the other NBD derivatives, NBDH, resulting in the solid SBA-NBDH.

### 3.4. Antioxidant Activity of the SBA-15-NBD Derivatives

The best-known method of determining the antioxidant activity of compounds is the use of 1,1-diphenyl-2-picrylhydrazil (DPPH) assay [35,41]. From the literature, it is known as DPPH is a stable free radical and has been used as a model compound to evaluate the effectiveness of antioxidants. For this, the absorbance of a freshly prepared methanolic solution of DPPH (0.1 mM) was measured at 515 nm using a UV–vis spectrophotometer. Then, 2 mL of 0.1 mM methanolic solution of DPPH was added over 5 mg of sample, stirred at room temperature and, the UV–vis spectrum of the resulting solution was recorded [35]. The percentage of inhibition of DPPH reduction was calculated according to Equation (1).

Inhibition (%) = [(A_0_ − A_30_)/A_0_] × 100 (1)

where A_0_ = absorbance at 0 min and A_30_ is absorbance at 30 min.

### 3.5. Measurement of Fluorescence Spectra

UV–vis absorption spectra of NBDH, NBD-PD, SBA-NBDH, and SBA-NBD-PD, in solid state/powder, were performed with a Perkin Elmer Lambda 35 spectrometer equipped with an integrating sphere, using a spectral on as a certified reflectance standard, at a scanning speed of 60 nm/min and a slit of 4 nm. Fluorescence emission and excitation spectra of NBDH, NBD-PD, SBA-NBDH, and SBA-NBD-PD, in solid state/powder, were registered using the Jasco FP-6500 spectrofluorometer, equipped with EFA-383 epifluorescence attachment, at a scanning speed of 100 nm/min and an excitation wavelength of 450 nm.

### 3.6. Biological Evaluation Procedures

Samples were incubated in standard conditions of temperature and humidity (37 ± 2 °C, 5 ± 1% CO_2_, and more than 90% humidity) for different time intervals (0, 4, 12, and 24 h, respectively). Following the incubation time, the samples were centrifuged at 20,000× *g* and the supernatant was collected for fluorescence intensity measurements registered for the maximum excitation and emission wavelengths of NBDH and NBD-PD.

#### 3.6.1. Cell Cultures

The biological evaluation was performed using the following cell lines: B16-F12 melanoma cells (ATCC CRL-6475, USA) and BJ human fibroblast cells (ATCC CRL-2522, USA), respectively. The cells were cultured in Dulbecco’s Modified Eagle’s Medium (DMEM) containing L-glutamine (Biochrom, Merck Millipore) and supplemented with 10% fetal bovine serum (FBS), 1% penicillin and streptomycin antibiotics in standard conditions of temperature and humidity (37 ± 2 °C, 5 ± 1% CO_2_ and more than 90% humidity).

The compounds were suspended in DMSO at a stock concentration of 10 mg/mL and then sterilized by Gamma irradiation. Afterwards, the compounds were suspended in complete culture medium (DMEM with 10% FBS fetal bovine serum) at concentrations of 50, 62.5, 100, 125, 250 and, 500 µg/mL, respectively. The NBDH and NBD-PD were dissolved in DMEM at equivalent concentrations to the nanoparticles, which were determined from the thermogravimetric analysis.

Two thousand cells/well were seeded in 96-well plates and incubated for 4 h in order to allow their attachment. The nanoparticle suspensions were added to the cells and incubated for another 16 h in standard conditions of temperature and humidity.

#### 3.6.2. Cytotoxicity and Proliferation Determination

The cellular viability and proliferation were quantitatively measured using the MTT tetrazolium-salt (y (3-(4,5-dimethylthiazol-2-yl)-2,5- diphenyltetrazolium bromide)) assay (Serva). For this, at the corresponding time-point, the medium was removed and gently replaced with fresh culture medium containing 10% MTT solution (5 mg/mL in PBS). The cells were incubated for another 3 h in standard conditions and afterwards the supernatant was replaced with DMSO, in order to solubilize the grown formazan crystals. The absorbance corresponding to each sample was measured at 570 nm wavelength using a Mithras 940 microplate reader (Berthold, Bad Wildbad, Germany). Cell viability was calculated as (Abs sample/Abs control) × 100. The half maximal inhibitory concentration (IC50) representing the concentration of the compound that reduces viability/proliferation to 50% of the control was determined by a sigmoidal fit using Origin Pro 8.1 (Microcal Inc., Northampton, MA, USA) software. All samples were performed in triplicate and each experiment was repeated 3 times. The selectivity index (SI) was calculated as follows: selectivity index (SI) = IC50 of normal cells/IC50 of tumoral cells.

#### 3.6.3. Cells Morphology, Cytoskeleton Evaluation and Nanoparticles Internalization

Additionally, cells were seeded onto 10 mm glass coverslips at a concentration of 10,000 cells/slide. The cells were incubated for 4 h, in order to allow their attachment and then were treated with nanoparticles at a concentration of 125 µg/mL and incubated for another 16 h. This timeframe is chosen because it is less than the doubling time of both cell lines and prevents any dilution of the nanoparticle intracellular content. Following this time, the cells were washed several times with PBS and fixed using 4% paraformaldehyde. Then, the actin filaments were stained using Texas Red Phalloidin and the nuclei using Hoechst. Fluorescence images were recorded using separate filters for the actin filaments, nuclei and respectively the functionalized nanoparticles. Then, the images were reconstructed using ImageJ software by overlapping.

#### 3.6.4. Clonogenic Assay

Clonogenic assay on 2D melanoma cells: the long-term survival was performed for B16-F12 melanoma cells at 10 days following treatment using the clonogenic assay. For this, the cells were cultivated and treated similarly to the MTT assay. After the cells incubation with the compounds, they were detached, counted, and seeded at a concentration of 1000 cells/well in 6-well plates. Then, the cells were incubated in standard conditions of temperature and humidity for 10 days. Afterwards, the cells were washed with PBS, fixed, and stained using a 0.5% Giemsa solution in methanol. Colonies containing more than 50 cells were counted. The plating efficiency was calculated, and the results were reported to control (untreated cells).

#### 3.6.5. Viability Assay on Melanoma Spheroids

Three-dimensional cell models of melanoma were obtained by seeding 10,000 spheroids in a low attachment round bottom 96-well spheroid plate (Corning). The spheroids were allowed 3 days to form and then the medium was collected and replaced with medium containing nanoparticles at the investigated concentrations. Then, the spheroids were incubated for another 5 days in presence of the nanoparticles. Following the incubation time, the cellular viability was assessed using the MTS [3-(4,5-dimethylthiazol-2-yl)-5-(3-carboxymethoxyphenyl)-2-(4-sulfophenyl)-2H-tetrazolium, inner salt; MTS] proliferation assay (CellTiter 96 Aqueous One Solution Cell Proliferation Assay, Promega), according to the producer specifications.

#### 3.6.6. Morphological Evaluation of Different Types of Cell Death (Apoptosis, Mitotic Catastrophe and Senescence)

The mitotic catastrophe assay was performed according to the protocol described by Popescu et al. [42] for nanoparticles. The effect was assessed at 5 days following the treatment. For this, 10,000 cells were seeded.

#### 3.6.7. Statistics

All experiments were performed in triplicate and the data were presented as the mean ± SEM. The statistical analysis was performed using a two-tailed Student’s test, where values of * *p* ≤ 0.05, ** *p* ≤ 0.01, *** *p* ≤ 0.001 were considered statistically significant.

## 4. Conclusions

In summary, in this work, we have reported the preparation of two novel fluorescent mesoporous silica composites through the covalent immobilization of [4-hydrazinyl-7-nitrobenz-[2,1,3-*d*]-oxadiazole (NBDH) and N1-(7-nitrobenzo[*c*][1,2,5] oxadiazol-4-yl)benzene-1,2-diamine (NBD-PD), respectively, on the surface of mesoporous silica SBA-15. The certification of obtaining the materials was made using different characterization techniques such as infrared, fluorescence, and X-ray photoelectron spectroscopy, thermal analysis, nitrogen physisorption, and scanning electron microscopy/transmission electron microscopy. Bioevaluation results suggest that both materials have antitumor potential on B16 melanoma cells. This claim is supported by the results that show a lower toxicity of these biomaterials on normal BJ cells compared to melanoma cells. In vitro tests showed that the newly synthesized materials decreased melanoma cell viability in a dose-dependent manner. Therefore, these results recommend the obtained biocomposites for oxidative stress tracking and amelioration, as well as for the development of novel antitumor theranostic platforms.

## Figures and Tables

**Table 1:** Textural parameters of the samples determined from the N_2_ adsorption–desorption experiments.

Sample	S_BET_ (m^2^g^−1^)	V_total_ (cm^3^g^−1^)	Average Pore Size (nm)
SBA-15	778.9	1.177	6.04
SBA-Cl	631.6	1.018	6.01
SBA-NBDH	562.0	0.944	6.04
SBA-NBD-PD	587.2	0.972	6.06

**Table 2:** Interplanar distances of the samples calculated from SAXS results.

Sample	d_100_ (nm)	d_110_ (nm)	d_200_ (nm)	d_201_ (nm)	d_300_ (nm)
SBA-15	10.4	5.9	5.2	3.8	3.4
SBA-NBDH	10.1	5.7	5.1	-	-
SBA-NBD-PD	10.4	5.9	5.2	-	-

**Table 3:** Surface composition of SBA functionalized matrix from XPS.

Sample	C1s (%)	O1s (%)	Si 2p(%)	N1s (%)	Cl 2p
SBA-15	3.4	85.76	10.77	-	-
SBA-Cl	4.27	84.54	10.55	-	0.64
SBA-NBDH	6.4	83.28	10.04	0.28	-
SBA-NBD-PD	8.74	81.32	9.5	0.44	-

**Table 4:** IC50 values of SBA-15-NBD derivates against BJ and B16 cells and their selectivity indexes.

Cells/IC50	IC50 (µg/mL) SBA-NBDH	IC50 (µg/mL) SBA-NBD-PD	Selectivity Index (SBA-NBDH)	Selectivity Index (SBA-NBD-PD)
BJ cells	138.63	136.13	-	-
B16 cells	120.12	114.11	1.15	1.19

## Data Availability

Not applicable.
